# Epigenome-wide association study in Chinese monozygotic twins identifies DNA methylation loci associated with blood pressure

**DOI:** 10.1186/s13148-023-01457-1

**Published:** 2023-03-03

**Authors:** Weijing Wang, Jie Yao, Weilong Li, Yili Wu, Haiping Duan, Chunsheng Xu, Xiaocao Tian, Shuxia Li, Qihua Tan, Dongfeng Zhang

**Affiliations:** 1 Department of Epidemiology and Health Statistics, Public Health College, Qingdao University, No. 308 Ningxia Road, Qingdao, 266021 Shandong China; 2 Jiangsu Health Development Research Center, Nanjing, Jiangsu China; 3 Population Research Unit, Faculty of Social Sciences, University of Helsinki, Helsinki, Finland; 4 Qingdao Municipal Center for Disease Control and Prevention/Qingdao Institute of Preventive Medicine, Qingdao, Shandong China; 5 Unit of Human Genetics, Department of Clinical Research, University of Southern Denmark, Odense, Denmark

**Keywords:** Blood pressure, Causality, DNA methylation, Monozygotic twins

## Abstract

**Background:**

Hypertension is a crucial risk factor for developing cardiovascular disease and reducing life expectancy. We aimed to detect DNA methylation (DNAm) variants potentially related to systolic blood pressure (SBP) and diastolic blood pressure (DBP) by conducting epigenome-wide association studies in 60 and 59 Chinese monozygotic twin pairs, respectively.

**Methods:**

Genome-wide DNA methylation profiling in whole blood of twins was performed using Reduced Representation Bisulfite Sequencing, yielding 551,447 raw CpGs. Association between DNAm of single CpG and blood pressure was tested by applying generalized estimation equation. Differentially methylated regions (DMRs) were identified by *comb-P* approach. Inference about Causation through Examination of Familial Confounding was utilized to perform the causal inference. Ontology enrichment analysis was performed using Genomic Regions Enrichment of Annotations Tool. Candidate CpGs were quantified using Sequenom MassARRAY platform in a community population. Weighted gene co-expression network analysis (WGCNA) was conducted using gene expression data.

**Results:**

The median age of twins was 52 years (95% range 40, 66). For SBP, 31 top CpGs (*p* < 1 × 10^–4^) and 8 DMRs were identified, with several DMRs within *NFATC1*, *CADM2*, *IRX1*, *COL5A1*, and *LRAT*. For DBP, 43 top CpGs (*p* < 1 × 10^–4^) and 12 DMRs were identified, with several DMRs within *WNT3A*, *CNOT10*, and *DAB2IP*. Important pathways, such as Notch signaling pathway, p53 pathway by glucose deprivation, and Wnt signaling pathway, were significantly enriched for SBP and DBP. Causal inference analysis suggested that DNAm at top CpGs within *NDE1*, *MYH11*, *SRRM1P2*, and *SMPD4* influenced SBP, while SBP influenced DNAm at CpGs within *TNK2*. DNAm at top CpGs within *WNT3A* influenced DBP, while DBP influenced DNAm at CpGs within *GNA14*. Three CpGs mapped to *WNT3A* and one CpG mapped to *COL5A1* were validated in a community population, with a hypermethylated and hypomethylated direction in hypertension cases, respectively. Gene expression analysis by WGCNA further identified some common genes and enrichment terms.

**Conclusion:**

We detect many DNAm variants that may be associated with blood pressure in whole blood, particularly the loci within *WNT3A* and *COL5A1*. Our findings provide new clues to the epigenetic modification underlying hypertension pathogenesis.

**Supplementary Information:**

The online version contains supplementary material available at 10.1186/s13148-023-01457-1.

## Introduction

Hypertension is a chronic disease condition, and approximately 90% of the cases are considered as essential hypertension without a definitive cause. The prevalence of hypertension is 28.9%, and the rates of treatment and control of hypertension are only 35.3% and 13.4% in China. Hypertension has been a considerable risk factor for developing cardiovascular disease and reducing life expectancy and has become a huge burden on the nationwide health system [1, 2].

As a complex phenotype, hypertension is controlled by both genetic and environmental factors through the interface of epigenetics. At present, the magnitude of genetic sources of variance in hypertension has been extensively explored. Wu et al. found that blood pressure had a moderate heritability with 0.61 for systolic blood pressure (SBP) and 0.58 for diastolic blood pressure (DBP) in Chinese population [3]. Additionally, genome-wide association studies (GWASs) have reported some genetic variants that were responsible for susceptibility to blood pressure variation, such as the genetic loci within *ADRB1*, *ATP2B1*, *SOX6*, *CHIC2*, *IGFBP3*, and *KCNK3* [4–8]. However, the previous reported genetic variants only partially contributed to the pathogenesis of hypertension.

In recent years, increasingly strong evidence has supported the significant role of epigenetic mechanisms with altered gene expression in the increased susceptibility to diseases. Currently, a large number of epigenome-wide association studies (EWASs) have been conducted to explore the underlying association between genomic DNA methylation (DNAm) variants and complex traits, such as heart failure [9] and permanent atrial fibrillation [10]. Meanwhile, accumulating evidence has also demonstrated a functional role of DNAm variants in the regulation of blood pressure or the development of hypertension [11, 12]. However, to date, very few studies have investigated the blood pressure or hypertension-related DNAm loci by applying an EWAS approach [13–16], and few results are replicated. In addition, the causal nature of the association, i.e., if DNAm exerts a causal effect on blood pressure or vice versa, is unknown. Hence, it is essential to further perform EWAS as well as causal inference analysis to investigate the association and causal relationship between DNAm and blood pressure.

Furthermore, the previous EWASs were most performed using samples from unrelated individuals, where the confounding effects from different genetic backgrounds were not well controlled for. Nowadays, the trait or disease-discordant twin design has been a popular and powerful tool for EWAS while controlling for individual genetic make-up [17, 18]. In this study based on a sample of blood pressure-discordant Chinese monozygotic twins, we conducted an EWAS to explore the association between the DNAm at CpGs and blood pressure as well as their causality and validated the candidate CpGs in a community population. Additionally, we further integrated the differentially methylated results with gene expression data.

## Materials and methods

The primary materials and methods of this study were in accord with our previously published studies [19–22].

### Participants

The sample collection was carried out through the Qingdao Twin Registry [23], and details of study recruitment have been previously described [24]. Participants who were pregnant and breastfeeding, who suffered from cardiovascular disease, stroke and/or tumor, and who were regularly taking any medications within one month before participation were excluded. Meanwhile, participants who were unconscious, unable, or unwilling to cooperate were also dropped. Considering that we used trait-discordant monozygotic twin design, the twins with intra-pair blood pressure difference ≥ 2 mmHg for SBP or intra-pair blood pressure difference ≥ 1 mmHg for DBP were separately chosen. A total of 60 SBP-discordant monozygotic twin pairs and 59 DBP-discordant twin pairs were included in the methylation analysis. The median of absolute values of intra-pair blood pressure difference was 18 mmHg (95% range 2, 55) for SBP and 10 mmHg (95% range 2, 28) for DBP, respectively. Additionally, a subsample of 12 monozygotic twin pairs were included in the gene expression analysis. All co-twin pairs completed a questionnaire and undertook a health examination after a 10–12-h overnight fast.

This study was approved by the Regional Ethics Committee of the Qingdao CDC Institutional Review Boards. The ethical principles of the Helsinki Declaration were also followed. Prior written informed consent was achieved.

### Zygosity determination

We first identified potential monozygotic and dizygotic twins through sex and ABO blood types. Twins with opposite sex and/or different blood types were classified as dizygotic twins. Then, the zygosity of twins with same sex and blood types was further determined by DNA testing using 16 short tandem repeat markers [23, 25, 26].

### Measurement of blood pressure

Blood pressure was measured in a sitting position following standard procedure using a mercurial table stand model sphygmomanometer. SBP was measured as Korotkoff phase I (appearance of sound) and DBP as Korotkoff phase V (disappearance of sound). Each subject took three repeated measurements, with at least one-minute interval. The mean value of these three measurements was calculated and used in subsequent analysis. All measurements greater than three standard deviations above or below the means were assigned as missing values.

### Reduced representation bisulfite sequencing (RRBS) data preparation

The total DNA extracted from whole blood was used in RRBS experiment. Briefly, genomic DNA was first digested to generate short fragments. Then the CpG-rich DNA fragments was bisulfite-converted. Finally, the cDNA library was obtained and sequenced. The raw methylation data covered 551,447 CpGs across the genome of each individual. We mapped the raw sequencing data to the human GRCh37 by *Bismark* [27] and then imported data to *BiSeq* to smooth the methylation level [28]. We controlled the coverage to 90% quantile and dropped CpGs with average methylation *β*-values less than 0.01 or more than 10 missing observations. After quality control, a total of 248,262 CpGs for SBP and 248,955 CpGs for DBP remained for subsequent analyses. The methylation *β*-value was transformed to *M*-value by applying log_2_ transformation.

Since total DNA was extracted from whole blood, different methylation profiles of distinct cell-types may lead to false discoveries [29]. In our analysis, we applied *ReFACTor* method, a reference-free method to account for cell-type heterogeneity, and we used the top five components to correct for the cell-type composition effect on DNAm [30].

### Gene expression data preparation

Briefly, the total mRNA was first extracted from whole blood. Subsequently, the RNA-Seq library was constructed and sequenced to obtain the sequenced data, which was then mapped to the human genome by *TopHat*_*2*_ [31]. The gene expression level was evaluated by FPKM value through *Cufflinks* software [32].

### Epigenome-wide association analysis

The association between the DNAm *M*-value at each CpG and SBP or DBP was tested by using generalized estimating equation (GEE) approach through the *geeglm* function in R-package *geepack*, adjusting for age, sex, and cell-type composition. Moreover, in order to address the paired structure of the twin data, we included a vector which identified the clusters of twins within a pair into the GEE model. To correct for multiple testing, we calculated false discovery rate (FDR) [33] and defined FDR < 0.05 as genome-wide significance. For CpGs with FDR ≥ 0.05, we defined *p* < 1 × 10^–6^ as suggestive significance and 1 × 10^–6^ ≤ *p* < 1 × 10^–5^ as weaker-than-suggestive significance [34]. The CpGs with *p* < 1 × 10^–4^ were reported as top CpGs of this EWAS [35]. The identified genomic CpGs (*p* < 0.05) were annotated to the nearest genes by using R-package *biomaRt* [36, 37].

### Causal inference analysis

For the top CpGs (*p* < 1 × 10^–4^), the causal relationship with blood pressure was investigated by the Inference about Causation through Examination of Familial Confounding (ICE FALCON) method which was a regression based method for causal inference in association studies using twins or family data [38, 39]. In this method, ‘familial’ meant both genetic and shared environmental factors in twins, which was essential to make explicit causal inference. The GEE model was applied for parameter estimation with correction for twin pairing. Estimations of *β*_self_, *β*_co-twin_ as well as *β’*_self_ and *β’*_co-twin_ were calculated, where *β*_self_ was the estimation of overall correlation including the causal proportion and family confounding proportion, *β*_co-twin_ estimated only the family confounding proportion of the correlation, and *β’*_self_ and *β’*_co-twin_ was the estimation of full model. If |*β*_co-twin_ *– β’*_co-twin_| was similar to |*β*_self_* – β’*_self_|, then the association was due to family confounding; and if |*β*_co-twin_ *– β’*_co-twin_| was much larger than |*β*_self_*—β’*_self_| (ratio > 1.5), then it indicated a causal effect.

### Region-based analysis

We applied the *comb-p* approach to detect the blood pressure-associated differentially methylated regions (DMRs) [40]. The significant enriched DMRs were determined by Stouffer–Liptak–Kechris (*slk*) corrected *p* < 0.05.

### Ontology enrichments analysis

We submitted the identified CpGs (*p* < 0.05) to the Genomic Regions Enrichment of Annotations Tool (GREAT) online to analyze the ontology enrichments [41]. Annotation was based on the human GRCh37, and the default “basal plus extension” association rule was used. The false discovery rate (FDR) < 0.05 was considered as statistically significant in ontology enrichments analysis.

### EWAS power estimation

We have recently published a computer simulation study on the power of EWAS using twin design [17]. According to this study, if one trait/disease had a heritability (*h*^2^) of 0.6 and there was a low correlation between environmental factors and DNAm (*R*^2^_*M,E*_ = 0.1), the sample size required for statistical power to exceed 80% in trait or disease-discordant twin design ranged from 22 (when the correlation within twin pair due to either shared genetic background or common environment, denoted as *ρ*_*ε*_ = 0.8) to 63 (when *ρ*_*ε*_ = 0.1) pairs, which was an immense improvement over the ordinary case–control design. Hence, we speculated that our study based on nearly 60 twin pairs would get a statistical power of about 80%.

We also estimated the correlation between environmental factors (i.e., blood pressure) and DNAm based on the top CpGs identified in this EWAS. We tested the correlation between intra-pair blood pressure difference and intra-pair DNAm difference of each top CpG in EWAS by using partial correlation analysis model, adjusting for age and sex. The median of absolute values of partial correlation coefficients was 0.34 (range 0.03, 0.47) for SBP and 0.27 (range 0.04, 0.46) for DBP (Additional file 1: Table S1), indicating that the *R*^2^_*M, E*_ of our study was likely to be greater than 0.1 and close to 0.3. The heritability of SBP and DBP was about 0.60 in the same twin population as our study [3]. According to the computer simulation study [17], for SBP and DBP with *h*^2^ = 0.6 and *R*^2^_*M, E*_ = 0.3, the sample size required for statistical power to exceed 80% in our twin design would range from 17 (when *ρ*_*ε*_ = 0.8) to 25 (when *ρ*_*ε*_ = 0.1) pairs, which were much less than 60 pairs. Hence, our study based on nearly 60 twin pairs would get an enough statistical power.

### Quantitative methylation analysis of COL5A1 and WNT3A

We randomly recruited 118 hypertension cases and 149 health controls from the community to validate the CpGs mapped to *COL5A1* and *WNT3A* in EWAS. The cases were defined as those with SBP ≥ 140 mmHg and DBP ≥ 90 mmHg. The subjects with a history of diabetes, obesity, cancer, stroke, and cardiovascular disease were excluded. The participants were interviewed when blood samples were taken and stored under − 80 °C for DNA methylation analysis. We designed the primers for *COL5A1* and *WNT3A* gene to cover the region with the most CpGs (*p* < 0.05) in EWAS. The mass spectra of cleavage products were collected using the MALDI-TOF mass spectrometry based on the MassARRAY System (Bio Miao Biological Technology, Beijing, China), and the spectra’s methylation ratio was generated by MassARRAY EpiTYPER software (Agena Bioscience, San Diego, California). The DNAm of CpGs between the two independent groups was compared by Wilcoxon rank-sum test. Binary logistic regression model was applied to evaluate the association of each CpG with hypertension while adjusting for BMI, triglyceride (TG), and fasting blood glucose (FBG). The *p* < 0.05 was set as statistically significant.

### Weighted gene co-expression network analysis (WGCNA)

We conducted the weighted gene co-expression network analysis (WGCNA) [42, 43] to identify the specific modules and genes potentially associated with blood pressure. Briefly, a weighted adjacency matrix using gene expression profile data was established, and then, a topological overlap matrix was constructed and used as input for hierarchical clustering analysis. Gene modules were detected by the dynamic tree cutting algorithm, and module eigengenes were correlated with SBP or DBP to detect the module of interest. Enrichment analysis was conducted for the genes clustered in the specific module by DAVID tool [44, 45]. The significant enriched terms were identified with *p* < 0.05 from a modified Fisher’s exact test.

## Results

### Epigenome-wide association analysis

A total of 60 twin pairs with a median value of 134.00 mmHg (95% range 102.05, 184.90) for SBP and 59 twin pairs with a median value of 80.00 mmHg (95% range 62.00, 105.03) for DBP were included in the methylation analysis. The median age of twins was 52 years (95% range 40, 66). The other clinical indicators, i.e., BMI, serum uric acid, FBG, high-density lipoprotein cholesterol (HDLC), low-density lipoprotein cholesterol (LDLC), and TG, showed statistically intra-pair correlated, indicating the co-twin design beneficial (Additional file 2: Table S2).

The Manhattan plot of EWAS on SBP is shown in Additional file 3: Fig. S1 (a), and we identified 31 SBP-related top CpGs with *p* < 1 × 10^–4^ (Table 1). After correcting for multiple testing, no CpG reached genome-wide significance as defined by FDR < 0.05. The four strongest associations (*β* = − 0.01, *p* = 5.76 × 10^–6^–9.58 × 10^–6^) were detected for the CpGs (chr3: 84,330,415–84,330,441 bp) located at *SRRM1P2*, showing weaker-than-suggestive significance (1 × 10^–6^ ≤ *p* < 1 × 10^–5^). All these top CpGs were located at/near 15 genes, including *SRRM1P2*, *COL5A1*, *MIR1268A*, *NFATC1*, *NDE1*, *MYH11*, *SMPD4*, *TXNL1P1*, *MIR3147*, *PIP5K1C*, *TNK2*, *CACHD1*, *SLC47A1*, etc.

**Table 1:** The results of epigenome-wide association study on systolic blood pressure (*p* < 1 × 10^–4^)

Chromosome	Position (bp)	Coefficient	*p*-value	FDR	Ensembl gene ID	HGNC symbol
chr3	84,330,432	− 0.009	5.756E-06	0.161	ENSG00000242195	*SRRM1P2*
chr3	84,330,437	− 0.009	7.677E-06	0.161	ENSG00000242195	*SRRM1P2*
chr3	84,330,415	− 0.009	8.172E-06	0.161	ENSG00000242195	*SRRM1P2*
chr3	84,330,441	− 0.009	9.579E-06	0.161	ENSG00000242195	*SRRM1P2*
chr3	84,330,448	− 0.009	1.248E-05	0.161	ENSG00000242195	*SRRM1P2*
chr17	58,216,280	− 0.008	1.285E-05	0.161	ENSG00000267095	NA
chr7	57,472,878	0.013	1.662E-05	0.196	ENSG00000266168	*MIR3147*
chr8^*^	9,260,932	− 0.076	2.635E-05	0.267	ENSG00000254235	NA
					ENSG00000254237	NA
chr17	58,216,262	− 0.008	2.687E-05	0.267	ENSG00000267095	NA
chr3	84,330,462	− 0.008	2.873E-05	0.272	ENSG00000242195	*SRRM1P2*
chr9	137,673,895	− 0.009	2.971E-05	0.272	ENSG00000130635	*COL5A1*
chr9	137,673,907	− 0.009	3.066E-05	0.272	ENSG00000130635	*COL5A1*
chr16^*^	15,814,807	− 0.048	3.425E-05	0.286	ENSG00000072864	*NDE1*
					ENSG00000133392	*MYH11*
chr13	87,444,790	− 0.011	3.545E-05	0.286	ENSG00000231879	*TXNL1P1*
chr18	77,269,485	0.011	4.124E-05	0.320	ENSG00000131196	*NFATC1*
chr9	137,673,888	− 0.009	4.699E-05	0.341	ENSG00000130635	*COL5A1*
chr13	87,444,783	− 0.011	5.913E-05	0.366	ENSG00000231879	*TXNL1P1*
chr15	22,545,461	0.007	5.953E-05	0.366	ENSG00000221641	*MIR1268A*
chr2	130,937,909	0.059	6.043E-05	0.366	ENSG00000136699	*SMPD4*
chr15	22,545,464	0.007	6.663E-05	0.384	ENSG00000221641	*MIR1268A*
chr8^*^	9,260,942	− 0.071	6.811E-05	0.384	ENSG00000254235	NA
					ENSG00000254237	NA
chr19	3,670,396	0.010	7.317E-05	0.399	ENSG00000186111	*PIP5K1C*
chr3	195,609,985	0.050	7.510E-05	0.399	ENSG00000061938	*TNK2*
chr2	130,937,907	0.055	7.555E-05	0.399	ENSG00000136699	*SMPD4*
chr1	64,880,619	− 0.108	7.798E-05	0.403	ENSG00000158966	*CACHD1*
chr17	19,436,923	− 0.028	8.193E-05	0.415	ENSG00000142494	*SLC47A1*
chr18	77,269,508	0.012	8.697E-05	0.428	ENSG00000131196	*NFATC1*
chr16^*^	15,814,759	− 0.056	8.814E-05	0.428	ENSG00000072864	*NDE1*
					ENSG00000133392	*MYH11*
chr18	77,269,476	0.011	8.970E-05	0.428	ENSG00000131196	*NFATC1*
chr15	22,545,472	0.008	9.480E-05	0.444	ENSG00000221641	*MIR1268A*
chr17	62,775,172	0.012	9.845E-05	0.450	ENSG00000215769	*ARHGAP27P1-BPTFP1-KPNA2P3*

NA, not available; FDR, false discovery rate

^*^The CpG sites annotated to two genes

The association between DNAm of 43 top CpGs and DBP reached *p* < 1 × 10^–4^ level (Additional file 3: Fig. S1 (b) and Table 2). There were four CpGs (chr1: 228,195,260–228,195,292 bp) within *WNT3A* and one CpG (chr1: 2,391,479 bp) within *PLCH2* detected as showing genome-wide significance (FDR < 0.05). Seven CpGs within *SIM1*, *PLCH2*, *ATXN7L3B*, and *LOC646588* showed weaker-than-suggestive significance with 1 × 10^–6^ ≤ *p* < 1 × 10^–5^. All the top CpGs were located at/near 16 genes, and there was more than one CpG located at/near genes *ATXN7L3B*, *DAB2IP*, *WNT3A*, *GNA14*, *EYS*, *KCNT1*, *LOC646588*, *MGEA5*, *PGR*, *PLCH2*, *SAE1*, and *SIM1*.

**Table 2:** The results of epigenome-wide association study on diastolic blood pressure (*p* < 1 × 10^–4^)

Chromosome	Position (bp)	Coefficient	*p*-value	FDR	Ensembl gene ID	HGNC symbol
chr1	228,195,277	0.028	5.764E-08	0.010	ENSG00000154342	*WNT3A*
chr1	228,195,289	0.029	1.291E-07	0.010	ENSG00000154342	*WNT3A*
chr1	2,391,479	− 0.020	1.540E-07	0.010	ENSG00000149527	*PLCH2*
chr1	228,195,292	0.029	1.633E-07	0.010	ENSG00000154342	*WNT3A*
chr1	228,195,260	0.029	2.857E-07	0.014	ENSG00000154342	*WNT3A*
chr6	100,909,431	0.026	2.450E-06	0.090	ENSG00000112246	*SIM1*
chr1	2,391,466	− 0.018	2.541E-06	0.090	ENSG00000149527	*PLCH2*
chr12	74,797,036	− 0.047	4.032E-06	0.125	ENSG00000253719	*ATXN7L3B*
chr6	100,909,425	0.025	5.889E-06	0.163	ENSG00000112246	*SIM1*
chr12	74,797,017	− 0.044	7.783E-06	0.185	ENSG00000253719	*ATXN7L3B*
chr7	25,898,451	0.020	8.166E-06	0.185	ENSG00000223561	*LOC646588*
chr12	74,797,049	− 0.037	9.188E-06	0.191	ENSG00000253719	*ATXN7L3B*
chr7	25,898,447	0.020	1.231E-05	0.236	ENSG00000223561	*LOC646588*
chr17	38,088,968	0.185	1.593E-05	0.257	ENSG00000264968	NA
chr6	66,373,850	0.055	1.624E-05	0.257	ENSG00000188107	*EYS*
chr12	74,797,053	− 0.035	1.720E-05	0.257	ENSG00000253719	*ATXN7L3B*
chr9	80,272,835	− 0.068	1.752E-05	0.257	ENSG00000156049	*GNA14*
chr1	228,195,243	0.029	1.988E-05	0.273	ENSG00000154342	*WNT3A*
chr9	80,272,842	− 0.066	2.086E-05	0.273	ENSG00000156049	*GNA14*
chr9	80,272,845	− 0.066	2.338E-05	0.288	ENSG00000156049	*GNA14*
chr6	66,373,857	0.053	2.469E-05	0.288	ENSG00000188107	*EYS*
chr9	80,272,847	− 0.065	2.663E-05	0.288	ENSG00000156049	*GNA14*
chr12	74,797,056	− 0.034	2.825E-05	0.293	ENSG00000253719	*ATXN7L3B*
chr9	138,637,356	− 0.013	4.158E-05	0.400	ENSG00000107147	*KCNT1*
chr19	35,324,068	0.168	4.340E-05	0.400	ENSG00000267767	*LINC01801*
chr19	47,635,288	− 0.021	4.779E-05	0.425	ENSG00000142230	*SAE1*
chr9	138,637,337	− 0.011	5.604E-05	0.462	ENSG00000107147	*KCNT1*
chr12	74,796,990	− 0.040	5.764E-05	0.462	ENSG00000253719	*ATXN7L3B*
chr17	38,088,944	0.171	5.910E-05	0.462	ENSG00000264968	NA
chr9	124,308,134	0.014	6.588E-05	0.462	ENSG00000136848	*DAB2IP*
chr9	124,308,131	0.014	6.979E-05	0.462	ENSG00000136848	*DAB2IP*
chr19	47,635,313	− 0.017	7.074E-05	0.462	ENSG00000142230	*SAE1*
chr5	30,864,593	0.145	7.349E-05	0.462	ENSG00000241668	*RPL19P11*
chr9	124,308,128	0.014	7.539E-05	0.462	ENSG00000136848	*DAB2IP*
chr11	100,999,098	0.026	8.179E-05	0.462	ENSG00000082175	*PGR*
chr16	8,619,841	0.014	8.388E-05	0.462	ENSG00000232258	*TMEM114*
chr9	124,308,155	0.013	8.503E-05	0.462	ENSG00000136848	*DAB2IP*
chr10	103,551,798	0.150	8.742E-05	0.462	ENSG00000198408	*OGA*
chr17	38,088,933	0.169	8.800E-05	0.462	ENSG00000264968	NA
chr11	100,999,104	0.025	8.815E-05	0.462	ENSG00000082175	*PGR*
chr9	124,308,115	0.014	9.133E-05	0.462	ENSG00000136848	*DAB2IP*
chr9	124,308,162	0.012	9.381E-05	0.462	ENSG00000136848	*DAB2IP*
chr10	103,551,806	0.148	9.390E-05	0.462	ENSG00000198408	*OGA*

NA, not available; FDR, false discovery rate

We found 21 common CpGs (*p* < 1 × 10^–3^) between SBP and DBP, and these CpGs were annotated at genes *CACNA1B*, *LARP4B*, *CSNK1G2*, *LOC646588*, *HES4*, *PPIAP45*, *GPX1*, *METRNL*, *ROBO3*, and *LINC00943*.

We also compared previously reported significant blood pressure or hypertension-associated differentially methylated genes in EWASs [13–15] with our results. We defined our genes where CpGs with *p* < 0.05 were located as supportive to the reported results. The genes *CDC42BPB*, *ALDH3B2*, *DAB2IP*, *SLC7A5*, *VPS25*, *SLC43A1*, *SKOR2*, *ATXN1*, *ZMIZ1*, and *CPT1A* for SBP and *MAN2A2*, *CFLAR*, *CPT1A*, *DAB2IP*, *SLC7A5*, *PHGDH*, *SKOR2*, and *ZMIZ1* for DBP could be replicated (Additional file 4: Table S3).

### Causal inference analysis

The results of causal inference on the top CpGs (*p* < 1 × 10^–4^) with SBP and DBP are provided in Table 3. Interestingly, a causal effect of DNAm to SBP was clearly supported for 9 CpGs located at/near *NDE1* and *MYH11*, *TXNL1P1*, *SMPD4*, *SRRM1P2*, *TNK2*, and *CACHD1*, respectively. Out of these 9 CpGs, a causal effect of SBP to DNAm of 4 CpGs located at/near *TXNL1P1* and *SMPD4* was also observed.

**Table 3:** Results of causal inference analysis for systolic blood pressure and diastolic blood pressure

CpG No.	Chromosome	Position	HGNC symbol	Methylation to blood pressure					Blood pressure to methylation				
				*β*_self_change_	*p*_self_change_	*β*_co-twin_change_	*p*_co-twin_change_	Absolute value of ratio	*β*_self_change_	*p*_self_change_	*β*_co-twin_change_	*p*_co-twin_change_	Absolute value of ratio
*SBP*													
CpG1	chr3	84,330,462	*SRRM1P2*	0.640	0.247	− 2.869	0.046	4.483	− 0.001	0.307	0.001	0.198	–
CpG2	chr16	15,814,807	*NDE1, MYH11*	0.086	0.287	− 0.355	< 0.001	4.136	− 0.004	0.405	0.008	0.210	–
CpG3	chr13	87,444,790	*TXNL1P1*	0.628	0.212	− 2.764	0.001	4.403	− 0.001	0.208	0.002	0.035	1.746
CpG4	chr13	87,444,783	*TXNL1P1*	0.540	0.288	− 2.704	0.002	5.006	− 0.001	0.240	0.002	0.045	1.820
CpG5	chr2	130,937,909	*SMPD4*	0.080	0.041	0.290	< 0.001	3.650	0.004	0.456	− 0.017	0.006	4.545
CpG6	chr3	195,609,985	*TNK2*	0.209	0.041	0.322	0.006	1.538	− 0.003	0.602	− 0.012	0.062	–
CpG7	chr2	130,937,907	*SMPD4*	0.132	0.005	0.359	< 0.001	2.728	0.001	0.789	− 0.017	0.002	12.877
CpG8	chr1	64,880,619	*CACHD1*	− 0.091	0.019	− 0.300	0.015	3.309	− 0.005	0.533	0.022	0.080	–
CpG9	chr16	15,814,759	*NDE1, MYH11*	0.082	0.262	− 0.421	< 0.001	5.111	− 0.006	0.284	0.010	0.128	–
*DBP*													
CpG1	chr1	228,195,277	*WNT3A*	− 0.745	0.102	− 2.312	< 0.001	3.102	− 0.001	0.538	− 0.003	0.353	–
CpG2	chr1	228,195,289	*WNT3A*	− 0.785	0.142	− 2.759	< 0.001	3.515	0.000	0.819	− 0.001	0.737	–
CpG3	chr1	228,195,292	*WNT3A*	− 0.787	0.157	− 2.875	< 0.001	3.655	0.000	0.936	0.000	0.907	–
CpG4	chr1	228,195,260	*WNT3A*	− 0.451	0.133	− 1.083	0.015	2.404	− 0.002	0.294	− 0.005	0.103	–
CpG5	chr7	25,898,451	*LOC646588*	− 0.111	0.864	− 1.332	0.040	11.959	0.001	0.623	0.003	0.217	–
CpG6	chr7	25,898,447	*LOC646588*	− 0.143	0.820	− 1.301	0.038	9.112	0.001	0.634	0.002	0.263	–
CpG7	chr6	66,373,850	*EYS*	0.143	0.700	− 0.402	0.317	–	0.012	0.094	0.038	< 0.001	3.234
CpG8	chr9	80,272,835	*GNA14*	− 0.078	0.761	0.121	0.670	–	0.011	0.062	− 0.021	0.010	1.877
CpG9	chr9	80,272,842	*GNA14*	− 0.098	0.724	0.156	0.608	–	0.011	0.070	− 0.021	0.007	2.013

DBP, diastolic blood pressure; SBP, systolic blood pressure

As for DBP, the causal effect of DNAm to DBP was obviously found for 6 CpGs, with 4 at *WNT3A* and 2 at *LOC646588*. A causal effect of DBP influencing DNAm was also observed for another 8 CpGs, with 4 CpGs at *GNA14*, 2 CpGs at *EYS*, 1 CpG at *SAE1*, and 1 CpG at *TMEM114*, respectively.

### Region-based analysis

**Fig. 1:**
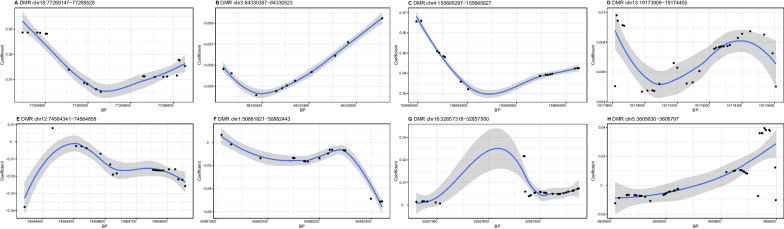
Differential methylation patterns from the identified differentially methylated regions for systolic blood pressure. The dots represent the CpGs. The *x*-axis shows the position of CpGs on chromosome and the *y*-axis shows regression coefficients. BP, base pair; DMR, differentially methylated region

A total of 8 DMRs were identified for SBP (Table 4). As illustrated in Fig. 1, the methylation levels of 4 DMRs (A, C, D, and G) at/near *NFATC1*, *LRAT*, *TUBA3C*, and *SLC6A10P* were positively and 3 DMRs (B, E, and F) at/near *CADM2*, *LOC100507377*, and *DMRTA2* negatively correlated with SBP, whereas the trend of association between one DMR (H) at *IRX1* and SBP was uncertain.

**Table 4:** Results of annotation to differentially methylated regions for systolic blood pressure and diastolic blood pressure

ID	Chromosome	Start (bp)	End (bp)	Length	Stouffer–Liptak–Kechris (*slk*) corrected *p*-value	Ensembl ID	Gene symbol
*SBP*							
A	chr18	77,269,147	77,269,528	20	< 0.001	ENSG00000131196	*NFATC1*
B	chr3	84,330,387	84,330,523	11	0.001	ENSG00000175161	*CADM2*
C	chr4	155,665,297	155,665,627	16	0.007	ENSG00000121207	*LRAT*
D	chr13	19,173,908	19,174,405	29	0.009	ENSG00000198033	*TUBA3C*
E	chr12	74,564,341	74,564,858	20	0.010	ENSG00000251138	*LOC100507377*
F	chr1	50,881,821	50,882,443	18	0.025	ENSG00000142700	*DMRTA2*
G	chr16	32,857,318	32,857,950	31	0.031	ENSG00000214617	*SLC6A10P*
H	chr5	3,605,630	3,606,797	44	0.031	ENSG00000170549	*IRX1*
*DBP*							
A	chr1	228,195,226	228,195,293	6	< 0.001	ENSG00000154342	*WNT3A*
B	chr20	21,376,425	21,376,894	28	0.003	ENSG00000125816	*NKX2-4*
C	chr16	8,619,759	8,619,952	10	0.006	ENSG00000232258	*TMEM114*
D	chr14	64,965,186	64,965,446	11	0.009	ENSG00000089775	*ZBTB25*
E	chr3	32,822,274	32,822,412	13	0.012	ENSG00000182973	*CNOT10*
F	chr17	38,088,678	38,088,969	11	0.018	ENSG00000172057	*ORMDL3*
G	chr19	47,933,149	47,933,251	4	0.027	ENSG00000118160	*SLC8A2*
H	chr19	1,465,543	1,467,185	80	0.029	ENSG00000115266	*APC2*
I	chr7	25,898,313	25,898,710	24	0.030	ENSG00000050344	*NFE2L3*
J	chr1	1,872,273	1,872,775	18	0.030	ENSG00000142609	*CFAP74*
K	chr9	124,308,098	124,308,286	11	0.031	ENSG00000136848	*DAB2IP*
L	chr18	14,999,329	15,000,083	47	0.040	ENSG00000180777	*ANKRD30B*

DBP, diastolic blood pressure; SBP, systolic blood pressure

**Fig. 2:**
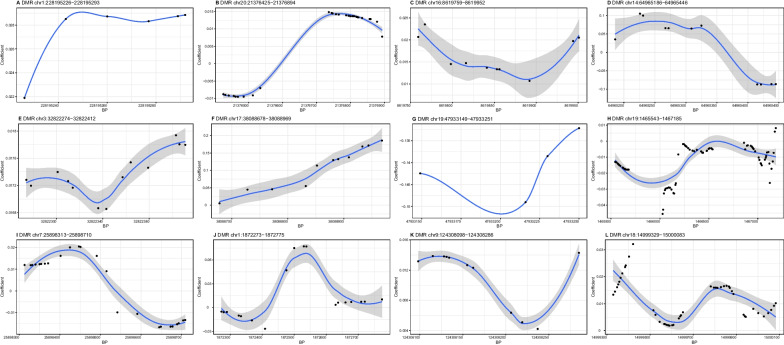
Differential methylation patterns from the identified differentially methylated regions for diastolic blood pressure. The dots represent the CpGs. The *x*-axis shows the position of CpGs on chromosome and the *y*-axis shows regression coefficients. BP, base pair; DMR, differentially methylated region

Out of the 12 DMRs identified for DBP (Fig. 2; Table 4), the methylation level of 6 DMRs (A, C, E, F, K, and L) showed positive associations and two DMRs (G and H) showed negative associations with DBP. But it was difficult to determine the trend of association between 4 DMRs (B, D, I, and J) and DBP. These DMRs were annotated within 12 genes, such as *WNT3A*, *CNOT10*, and *DAB2IP*.

### Ontology enrichments analysis

Lots of important ontology enrichments potentially associated with SBP were found, such as nicotinic acetylcholine receptor signaling pathway, p53 pathway by glucose deprivation, notch signaling pathway, Hedgehog signaling pathway, and PI3 kinase pathway (Table 5). For DBP, the ontology enrichments mainly highlighted inflammation mediated by chemokine and cytokine signaling pathway, notch signaling pathway, angiogenesis, Wnt signaling pathway, TGF-beta signaling pathway, etc. (Table 6).

**Table 5:** The top GREAT ontology enrichments for regions potentially related to systolic blood pressure

Ontology database	Term name	Binom FDR Q-value	Binom region fold enrichment
PANTHER pathway	Cytoskeletal regulation by Rho GTPase	1.29E-17	2.19
PANTHER pathway	Nicotinic acetylcholine receptor signaling pathway	8.79E-14	1.83
PANTHER pathway	Metabotropic glutamate receptor group II pathway	5.05E-11	2.01
PANTHER pathway	GABA-B receptor II signaling	2.71E-10	1.97
PANTHER pathway	p53 pathway by glucose deprivation	6.02E-09	2.57
PANTHER pathway	Angiogenesis	1.50E-07	1.35
PANTHER pathway	Inflammation mediated by chemokine and cytokine signaling pathway	2.66E-07	1.37
PANTHER pathway	Endogenous cannabinoid signaling	2.98E-07	2.05
PANTHER pathway	Notch signaling pathway	6.81E-07	1.78
PANTHER pathway	Hedgehog signaling pathway	4.77E-06	1.94
PANTHER pathway	Thyrotropin-releasing hormone receptor signaling pathway	7.80E-06	1.54
PANTHER pathway	Gamma-aminobutyric acid synthesis	8.53E-06	3.62
PANTHER pathway	Nicotine pharmacodynamics pathway	1.43E-05	1.82
PANTHER pathway	Heterotrimeric G-protein signaling pathway-rod outer segment phototransduction	5.11E-05	1.74
PANTHER pathway	Heterotrimeric G-protein signaling pathway-Gq alpha and Go alpha-mediated pathway	7.86E-05	1.29
PANTHER pathway	Corticotropin-releasing factor receptor signaling pathway	7.85E-04	1.68
PANTHER pathway	Adrenaline and noradrenaline biosynthesis	2.65E-03	1.67
PANTHER pathway	Insulin/IGF pathway-mitogen activated protein kinase kinase/MAP kinase cascade	5.49E-03	1.42
PANTHER pathway	PI3 kinase pathway	1.32E-02	1.34
PANTHER pathway	Histamine H1 receptor-mediated signaling pathway	1.97E-02	1.33
MSigDB pathway	Focal adhesion	1.43E-17	1.51
MSigDB pathway	Type II diabetes mellitus	2.42E-14	1.95
MSigDB pathway	Taurine and hypotaurine metabolism	8.37E-13	3.96
MSigDB pathway	RAC1 signaling pathway	1.00E-12	2.05
MSigDB pathway	Insulin signaling pathway	8.51E-10	1.49
MSigDB pathway	Regulation of RhoA activity	6.87E-09	1.82
MSigDB pathway	Arachidonic acid metabolism	7.32E-08	2.01
MSigDB pathway	T cell receptor signaling pathway	8.83E-06	1.39
MSigDB pathway	mTOR signaling pathway	7.31E-05	1.53
MSigDB pathway	VEGF signaling pathway	5.69E-04	1.42

**Table 6:** The top GREAT ontology enrichments for regions potentially related to diastolic blood pressure

Ontology database	Term name	Binom FDR Q-value	Binom region fold enrichment
PANTHER pathway	Inflammation mediated by chemokine and cytokine signaling pathway	1.42E-12	1.50
PANTHER pathway	Thyrotropin-releasing hormone receptor signaling pathway	9.07E-12	1.82
PANTHER pathway	Nicotine pharmacodynamics pathway	2.13E-11	2.27
PANTHER pathway	Endogenous cannabinoid signaling	2.44E-11	2.35
PANTHER pathway	Cytoskeletal regulation by Rho GTPase	2.68E-10	1.83
PANTHER pathway	Notch signaling pathway	1.52E-09	1.93
PANTHER pathway	Histamine H1 receptor-mediated signaling pathway	2.86E-09	1.82
PANTHER pathway	Muscarinic acetylcholine receptor 1 and 3 signaling pathway	6.39E-09	1.64
PANTHER pathway	Angiogenesis	4.17E-08	1.35
PANTHER pathway	2-Arachidonoylglycerol biosynthesis	1.35E-07	3.31
PANTHER pathway	Nicotinic acetylcholine receptor signaling pathway	7.36E-07	1.52
PANTHER pathway	Corticotropin-releasing factor receptor signaling pathway	1.51E-06	1.94
PANTHER pathway	p53 pathway by glucose deprivation	2.02E-06	2.21
PANTHER pathway	Heterotrimeric G-protein signaling pathway-rod outer segment phototransduction	2.16E-06	1.83
PANTHER pathway	Angiotensin II-stimulated signaling through G proteins and beta-arrestin	6.23E-06	1.79
PANTHER pathway	Wnt signaling pathway	7.41E-05	1.17
PANTHER pathway	GABA-B receptor II signaling	1.80E-04	1.54
PANTHER pathway	Blood coagulation	1.64E-03	1.64
PANTHER pathway	Insulin/IGF pathway-mitogen activated protein kinase kinase/MAP kinase cascade	5.21E-03	1.40
PANTHER pathway	TGF-beta signaling pathway	7.04E-03	1.22
PANTHER pathway	Beta3 adrenergic receptor signaling pathway	2.62E-02	1.46
PANTHER pathway	PI3 kinase pathway	3.04E-02	1.28
PANTHER pathway	FGF signaling pathway	3.54E-02	1.16
PANTHER pathway	Hedgehog signaling pathway	3.99E-02	1.40
PANTHER pathway	Toll receptor signaling pathway	4.29E-02	1.30
MSigDB pathway	Ceramide signaling pathway	1.96E-17	2.27
MSigDB pathway	RhoA signaling pathway	1.56E-13	2.05
MSigDB pathway	p53 pathway	3.68E-09	1.81
MSigDB pathway	VEGF signaling pathway	5.46E-06	1.54
MSigDB pathway	Insulin signaling pathway	3.20E-03	1.23

Many common ontology enrichments for SBP and DBP were observed, such as nicotinic acetylcholine receptor signaling pathway, p53 pathway by glucose deprivation, Notch signaling pathway, Hedgehog signaling pathway, and PI3 kinase pathway (Additional file 5: Table S4).

We found that 2 pathways (PKA-mediated phosphorylation of CREB, regulation of insulin secretion) for SBP and 2 pathways (NCAM1 interactions, dorso-ventral axis formation) for DBP were also enriched in our previous GWAS of blood pressure in twins [8].

### Quantitative methylation analysis of COL5A1 and WNT3A

Eight CpGs (*p* < 0.05) mapped to *COL5A1* in EWAS of SBP were quantified using the Sequenom MassARRAY platform. As shown in Additional file 6: Table S5, just one CpG (Chr9: 137,673,907) was validated to be hypomethylated (*β* = -0.439, *p* = 0.048) in hypertension cases, and this CpG was also regarded as top signal as in Table 1.

Among the 5 CpGs (*p* < 0.05) mapped to *WNT3A* in EWAS of DBP, 3 were quantified using the Sequenom MassARRAY platform. As shown in Additional file 7: Table S6, all of the 3 CpGs were validated in the same direction as in EWAS and also regarded as top signal as in Table 2. Overall, the validation analysis showed clear consistency of hypermethylation in 3 CpGs within *WNT3A* associated with DBP in a community population.

### Weighted gene co-expression network analysis (WGCNA) and gene expression analysis

We included 12 twin pairs (including 7 male pairs) with a median age of 53 years (95% range 43–65), a median SBP of 126 mmHg (95% range 94–195), and a median DBP of 81 mmHg (95% range 64–100) in the analyses.

Additional file 8: Fig. S2 illustrates the genes clustered in mediumpurple3 module (including 4,380 genes) were both negatively correlated with SBP (*r* =  − 0.45, *p* = 0.03) and DBP (*r* =  − 0.45, *p* = 0.03). Among the genes where the top CpGs (*p* < 1 × 10^–4^) and DMRs were annotated in methylation analysis, 3 genes (*MYH11*, *NFATC1*, and *PIP5K1C*) for SBP and 7 genes (*WNT3A*, *EYS*, *GNA14*, *SAE1*, *CNOT10*, *APC2*, and *CFAP74*) for DBP were also clustered in mediumpurple3 module in WGCNA.

The genes in methylation analysis and genes clustered in mediumpurple3 module were involved in some common enrichment terms, such as voltage-gated calcium channel activity, NADH dehydrogenase (ubiquinone) activity, PPAR signaling pathway, and acetylcholine receptor activity (Additional file 9: Table S7).

## Discussion

It has been demonstrated that epigenetics plays a crucial part in the development hypertension; hence, looking for the specific DNAm variants potentially associated with blood pressure is still a research hotspot [46]. In this study, we detected multiple CpGs, genes, DMRs, and pathways that could not only elucidate the mechanisms of blood pressure variation but also have important implications for the intervention and treatment of hypertension.

In our EWAS on SBP, many genes where the top CpGs and DMRs were located, such as *SRRM1P2*, *COL5A1*, *NFATC1*, *NDE1*, *MYH11*, *SMPD4*, *LRAT*, *CADM2*, *IRX1*, and *TNK2*, may play important roles in regulating blood pressure. The SNP rs6794880 (chr3:84,402,361) in *SRRM1P2* was reported to be related to obesity [47], and we suspected that this locus might influence the development of obesity through regulating the DNAm at one CpG (chr3:84,330,462) in *SRRM1P2* we identified. Moreover, the association between obesity and hypertension has clearly been confirmed [48]. It was indicated that the SNPs rs4841895 in *COL5A1* [49], rs4799055 in *NFATC1* (from dbGaP database), rs1449386 in *CADM2* [50], and rs954767 in *IRX1* [51] might play a role in blood pressure regulation, and we suspected that these loci might influence the development of hypertension through regulating the DNAm in these genes. *NDE1* gene was involved in the signaling pathway by Rho GTPases, which could play a critical role in the pathogenesis of hypertension [52]. The protein encoded by *MYH11* is a smooth muscle myosin in vascular smooth muscle cell (SMC) whose principal functions were contraction and regulation of blood pressure and blood flow distribution. The DNAm variation of *MYH11* might influence the function of SMC and hence took part in the pathogenesis of hypertension [53]. The protein encoded by *SMPD4* was a sphingomyelinase involved in sphingolipid metabolism pathway, and mounting evidence pointed toward that a derangement of this pathway might trigger the precursor clinical conditions of hypertension and hypertension itself [54]. It was found that *LRAT* may be a critical biomarker of vitamin A deficiency in target organs and may regulate blood pressure through affecting renin angiotensin system biomarkers [55]. *TNK2* gene was involved in the oxidative damage response pathway, and it was demonstrated that inflammation and oxidative stress significantly contributed to the vascular dysfunction and renal damage associated with hypertension [56]. However, the mechanism of other genes, such as *TXNL1P1*, *PIP5K1C*, *MIR3147*, and *SLC47A1*, underlining hypertension requires further investigation.

As for DBP, several interesting genes were also found, including *DAB2IP*, *WNT3A*, *GNA14*, *KCNT1*, *PGR*, *PLCH2*, *SIM1*, and *CNOT10*. It was previously reported that the SNPs rs35061590 and rs13290547 in *DAB2IP* might be associated with heart rate [57] and hence might influence the pathogenesis of hypertension. *WNT3A* gene was a member of the WNT gene family, and Wnt signaling pathway played an emerging role in regulating blood pressure [58]. The protein encoded by *GNA14* was involved in the regulation of insulin secretion pathway, and the relationship of insulin, insulin sensitivity, and hypertension had been clearly confirmed [59]. It was reported that the genetic knockout mouse strain lacking K_Na_ channels (*KCNT1* and *KCNT2*) showed a modest hypertensive phenotype [60]. The SNP rs61892344 in *PGR* was previously reported to be associated with DBP [51]. The protein encoded by *PLCH2* was involved in the inositol phosphate metabolism pathway, and the inositol phosphate production in blood vessels differed in normotensive and spontaneously hypertensive rats [61]. An association of *SIM1* variants with early-onset obesity in children was demonstrated [62], but the association of *SIM1* with hypertension was currently unknown. The *CNOT10* gene was probably associated with left ventricular remodeling in hypertension by bioinformatics-based analysis [63]. Up until now, the association of other genes, such as *ATXN7L3B*, *LOC646588*, *EYS*, *MGEA5*, and *SAE1*, with hypertension had not been extensively researched, but they may also serve as candidates to be further verified.

There is a particular challenge regarding the causal effects in observational epidemiological studies using high-dimensional omics data [64]. Our study provides evidence for the causation underlying the blood pressure–DNA methylation association using ICE FALCON method. We found the causal effect that SBP was in response to the DNAm at CpGs located at several genes. *NDE1* and *MYH11* were involved in the Rho GTPase effectors pathway whose important role in the pathogenesis of vasospasm, hypertension, pulmonary hypertension, and heart failure had been demonstrated [65]. *TNK2* was involved in the oxidative damage response pathway that could cause vascular dysfunction and renal damage associated with hypertension [56]. As for DBP, clear causal effect from DNAm to DBP was found for CpGs within *WNT3A* and *LOC646588*. *WNT3A* was involved in Wnt signaling pathway whose role in regulating blood pressure had previously been reported [58]. However, the mechanism of DNAm variation response to blood pressure changes was currently unclear, and further research was needed.

As additional validation, we quantified candidate CpGs mapped to *WNT3A* and *COL5A1* using Sequenom MassARRAY platform in a community population, and three CpGs mapped to *WNT3A* and one CpG mapped to *COL5A1* were successfully validated. As additional replication, we also compared previously reported results in EWASs with ours. A list of differentially methylated genes could be replicated, especially the well-known hypertension-associated gene *DAB2IP* [57]. We also compared the results from methylation and gene expression analyses and found a list of common genes. For SBP, these genes were involved in various biological pathways, such as nicotinic acetylcholine receptor signaling pathway (*MYH11*), Wnt signaling pathway (*NFATC1*), and RhoA signaling pathway (*PIP5K1C*). For DBP, these common genes took part in Wnt signaling pathway (*WNT3A*, *APC2*, and *GNA14*), ubiquitin proteasome pathway (*SAE1*), and RNA degradation pathway (*CNOT10*), etc. Moreover, we also found many common enrichment terms, such as voltage-gated calcium channel activity [66], NADH dehydrogenase (ubiquinone) activity [67], PPAR signaling pathway [68], and acetylcholine receptor activity [69], for which the relationships with hypertension were clear. All of these indicated that the DNAm variants we identified Additional file 8 played a significant role in the development of hypertension.

Several strengths can be noticed in our study. First, the trait or disease-discordant twin design we adopted has been revealed as a powerful tool for detecting the epigenetic variation underling complex diseases [18]. Second, we also performed causal inference to investigate the causation underlying the cross-sectional epigenetic associations and found that blood pressure changes had a causal effect on the DNAm variants at some CpGs, and vice versa. Third, given the various genetic constitutions, environmental exposures, and a multitude of lifestyles in different ethnic populations worldwide, our findings will specifically help elucidate the underlying pathogenesis of hypertension in the Chinese population.

Nevertheless, the sample size of the present study was relatively limited due to the challenges of recruiting and identifying qualified twins. However, the trait or disease-discordant twin design we adopted had greater statistical power over the traditional cross-sectional or case–control design. For blood pressure with a moderate heritability, this design would allow for large sample size reductions comparing to the traditional designs. According to our previous study [17], this study based on nearly 60 twin pairs would get a statistical power of about 80%.

## Conclusions

In summary, we found evidence that in peripheral blood from middle and old-aged Chinese twins, the DNAm at several loci within *WNT3A* and *COL5A1* is associated with blood pressure. Additionally, we also found evidence that blood pressure has a causal effect on peripheral blood DNAm, and vice versa. Our findings provide new clues to the epigenetic modification underlying hypertension pathogenesis.

## Supplementary Information

**Additional file 1: Table S1**. The results of partial correlation analysis model between intra-pair blood pressure difference and intra-pair DNA methylation difference of each top CpG in epigenome-wide association analysis

**Additional file 2: Table S2**. Basic characteristics of the participants

**Additional file 3: Fig. S1**. Circular Manhattan plot for epigenome-wide association studies of systolic blood pressure (a) and diastolic blood pressure (b). The numbers of chromosome and the -log10 of p-values for statistical significance are shown. The dots represent the observed CpGs.

**Additional file 4: Table S3**. Comparison between our results and other previously reported blood pressure or hypertension-associated differentially methylated genes

**Additional file 5: Table S4**. Common ontology enrichments by GREAT tool between systolic blood pressure and diastolic blood pressure

**Additional file 6: Table S5**. The results of validation analysis for the CpGs mapped to COL5A1 on systolic blood pressure

**Additional file 7: Table S6**. The results of validation analysis for the CpGs mapped to WNT3A on diastolic blood pressure

**Additional file 8: Fig. S2**. Relationships of consensus module eigengenes and external trait of blood pressure. Numbers in the table report the correlations with the p-values printed in parentheses. The table is color coded by correlation according to the color legend.

**Additional file 9: Table S7**. Common enrichment terms for blood pressure between methylation analysis and weighted gene co-expression network analysis

## Data Availability

The data used or analyzed during the current study are available from the corresponding author on reasonable request.

## Abbreviations

ICE FALCON: Causation through examination of familial confounding
DMR: Differentially methylated region
DBP: Diastolic blood pressure
EWAS: Epigenome-wide association study
FDR: False discovery rate
GREAT: Genomic Regions Enrichment of Annotations Tool
GEE: Generalized estimating equation
RRBS: Reduced-representation bisulfite sequencing
SBP: Systolic blood pressure
WGCNA: Weighted gene co-expression network analysis
