# Supplementary material for: Epigenome-wide association study in Chinese monozygotic twins identifies DNA methylation loci associated with blood pressure
Source: Clin Epigenetics. 2023 Mar 3;15:38. doi: 10.1186/s13148-023-01457-1 (PMC9985232; doi:10.1186/s13148-023-01457-1)
Supplement: Supplementary file 1 — Additional file 1: Table S1. The results of partial correlation analysis model between intra-pair blood pressure difference and intra-pair DNA methylation difference of each top CpG in epigenome-wide association analysis [file 13148_2023_1457_MOESM1_ESM.docx]

**Additional file 1: Table S1**. The results of partial correlation analysis model between intra-pair blood pressure difference and intra-pair DNA methylation difference of each top CpG in epigenome-wide association analysis

| **CpG** | |  | ***r*** | ***p*-value** |
| --- | --- | --- | --- | --- |
| **Chromosome** | **Position (bp)** |  |  |  |
| ***SBP*** |  |  |  |  |
| chr3 | 84,330,432 |  | -0.469 | < 0.001 |
| chr3 | 84,330,437 |  | -0.459 | < 0.001 |
| chr3 | 84,330,415 |  | -0.472 | < 0.001 |
| chr3 | 84,330,441 |  | -0.452 | < 0.001 |
| chr3 | 84,330,448 |  | -0.439 | 0.001 |
| chr17 | 58,216,280 |  | -0.274 | 0.037 |
| chr7 | 57,472,878 |  | 0.414 | 0.001 |
| chr8 | 9,260,932 |  | -0.333 | 0.011 |
| chr17 | 58,216,262 |  | -0.267 | 0.043 |
| chr3 | 84,330,462 |  | -0.428 | 0.001 |
| chr9 | 137,673,895 |  | -0.066 | 0.624 |
| chr9 | 137,673,907 |  | -0.034 | 0.797 |
| chr16 | 15,814,807 |  | -0.391 | 0.002 |
| chr13 | 87,444,790 |  | -0.423 | 0.001 |
| chr18 | 77,269,485 |  | 0.054 | 0.685 |
| chr9 | 137,673,888 |  | -0.078 | 0.560 |
| chr13 | 87,444,783 |  | -0.422 | 0.001 |
| chr15 | 22,545,461 |  | 0.341 | 0.009 |
| chr2 | 130,937,909 |  | 0.139 | 0.298 |
| chr15 | 22,545,464 |  | 0.341 | 0.009 |
| chr8 | 9,260,942 |  | -0.324 | 0.013 |
| chr19 | 3,670,396 |  | 0.420 | 0.001 |
| chr3 | 195,609,985 |  | 0.220 | 0.096 |
| chr2 | 130,937,907 |  | 0.120 | 0.370 |
| chr1 | 64,880,619 |  | -0.382 | 0.003 |
| chr17 | 19,436,923 |  | -0.067 | 0.619 |
| chr18 | 77,269,508 |  | 0.055 | 0.681 |
| chr16 | 15,814,759 |  | -0.389 | 0.003 |
| chr18 | 77,269,476 |  | 0.056 | 0.674 |
| chr15 | 22,545,472 |  | 0.339 | 0.009 |
| chr17 | 62,775,172 |  | 0.311 | 0.017 |
| ***DBP*** |  |  |  |  |
| chr1 | 228,195,277 |  | 0.245 | 0.066 |
| chr1 | 228,195,289 |  | 0.254 | 0.056 |
| chr1 | 2,391,479 |  | -0.055 | 0.682 |
| chr1 | 228,195,292 |  | 0.257 | 0.053 |
| chr1 | 228,195,260 |  | 0.202 | 0.133 |
| chr6 | 100,909,431 |  | 0.208 | 0.120 |
| chr1 | 2,391,466 |  | -0.044 | 0.744 |
| chr12 | 74,797,036 |  | -0.152 | 0.258 |
| chr6 | 100,909,425 |  | 0.199 | 0.137 |
| chr12 | 74,797,017 |  | -0.131 | 0.333 |
| chr7 | 25,898,451 |  | 0.300 | 0.023 |
| chr12 | 74,797,049 |  | -0.164 | 0.223 |
| chr7 | 25,898,447 |  | 0.289 | 0.029 |
| chr17 | 38,088,968 |  | 0.290 | 0.029 |
| chr6 | 66,373,850 |  | 0.178 | 0.186 |
| chr12 | 74,797,053 |  | -0.163 | 0.225 |
| chr9 | 80,272,835 |  | -0.465 | < 0.001 |
| chr1 | 228,195,243 |  | 0.149 | 0.267 |
| chr9 | 80,272,842 |  | -0.457 | < 0.001 |
| chr9 | 80,272,845 |  | -0.455 | < 0.001 |
| chr6 | 66,373,857 |  | 0.168 | 0.212 |
| chr9 | 80,272,847 |  | -0.451 | < 0.001 |
| chr12 | 74,797,056 |  | -0.161 | 0.232 |
| chr9 | 138,637,356 |  | -0.287 | 0.030 |
| chr19 | 35,324,068 |  | 0.222 | 0.098 |
| chr19 | 47,635,288 |  | -0.266 | 0.045 |
| chr9 | 138,637,337 |  | -0.266 | 0.046 |
| chr12 | 74,796,990 |  | -0.107 | 0.430 |
| chr17 | 38,088,944 |  | 0.265 | 0.046 |
| chr9 | 124,308,134 |  | 0.329 | 0.012 |
| chr9 | 124,308,131 |  | 0.328 | 0.013 |
| chr19 | 47,635,313 |  | -0.258 | 0.053 |
| chr5 | 30,864,593 |  | 0.280 | 0.035 |
| chr9 | 124,308,128 |  | 0.326 | 0.013 |
| chr11 | 100,999,098 |  | 0.298 | 0.024 |
| chr16 | 8,619,841 |  | 0.225 | 0.093 |
| chr9 | 124,308,155 |  | 0.333 | 0.011 |
| chr10 | 103,551,798 |  | 0.287 | 0.031 |
| chr17 | 38,088,933 |  | 0.263 | 0.048 |
| chr11 | 100,999,104 |  | 0.294 | 0.027 |
| chr9 | 124,308,115 |  | 0.323 | 0.014 |
| chr9 | 124,308,162 |  | 0.339 | 0.010 |
| chr10 | 103,551,806 |  | 0.289 | 0.029 |

**Note**: DBP, diastolic blood pressure; SBP, systolic blood pressure
