# Supplementary material for: Epigenome-wide association study in Chinese monozygotic twins identifies DNA methylation loci associated with blood pressure
Source: Clin Epigenetics. 2023 Mar 3;15:38. doi: 10.1186/s13148-023-01457-1 (PMC9985232; doi:10.1186/s13148-023-01457-1)
Supplement: Supplementary file 2 — Additional file 2: Table S2. Basic characteristics of the participants [file 13148_2023_1457_MOESM2_ESM.docx]

**Additional file 2: Table S2**. Basic characteristics of the participants

| **Characteristics** | **SBP** | | |  | **DBP** | | |
| --- | --- | --- | --- | --- | --- | --- | --- |
|  | **Values** | **Intra-pair correlation** | |  | **Values** | **Intra-pair correlation** | |
|  |  | ***r*** | ***p*-value** |  |  | ***r*** | ***p*-value** |
| Number of twin pairs | 60 |  |  |  | 59 |  |  |
| Gender, pairs (%) |  |  |  |  |  |  |  |
| Male | 32 (53) | **-** | **-** |  | 31 (53) |  |  |
| Female | 28 (47) | **-** | **-** |  | 28 (47) |  |  |
| Age, Median (P_2.5_, P_97.5_), years | 52 (40, 66) | **-** | **-** |  | 52 (40, 66) |  |  |
| BMI, mean (SD), kg/m^2^ | 24.92 (3.49) | 0.571 | < 0.001 |  | 24.89 (3.46) | 0.564 | < 0.001 |
| SBP/DBP, Median (P_2.5_, P_97.5_), mmHg | 134.00 (102.05, 184.90) | 0.408 | 0.001 |  | 80.00 (62.00, 105.03) | 0.177 | 0.181 |
| SUA, mean (SD)/ Median (P_2.5_, P_97.5_), μmol/L | 292.60 (93.57) | 0.427 | 0.001 |  | 277.50 (152.63, 521.68) | 0.531 | < 0.001 |
| FBG, Median (P_2.5_, P_97.5_), mmol/L | 5.40 (3.60, 10.73) | 0.608 | < 0.001 |  | 5.41 (3.60, 10.81) | 0.654 | < 0.001 |
| CHOL, mean (SD), mmol/L | 4.96 (1.21) | 0.569 | < 0.001 |  | 4.90 (1.18) | 0.548 | < 0.001 |
| TG, Median (P_2.5_, P_97.5_, mmol/L | 1.15 (0.20, 5.65) | 0.604 | < 0.001 |  | 1.12 (0.32, 5.66) | 0.582 | < 0.001 |
| HDLC, Median (P_2.5_, P_97.5_), mmol/L | 1.31 (0.67, 2.56) | 0.769 | < 0.001 |  | 1.36 (0.67, 2.71) | 0.771 | < 0.001 |
| LDLC, mean (SD), mmol/L | 2.92 (0.90) | 0.494 | < 0.001 |  | 2.85 (0.86) | 0.466 | < 0.001 |

**Note**: Continuous variables were presented as mean (standard deviation (SD)) or median (P_2.5_, P_97.5_); Categorical variables were presented as numbers with percentages

BMI, body mass index; CHOL, total cholesterol; DBP, diastolic blood pressure; FBG, fasting blood glucose; HDLC, high-density lipoprotein cholesterol; LDLC, low-density lipoprotein cholesterol; SBP, systolic blood pressure; SUA, serum uric acid; TG, triglyceride
