# Supplementary material for: Epigenome-wide association study in Chinese monozygotic twins identifies DNA methylation loci associated with blood pressure
Source: Clin Epigenetics. 2023 Mar 3;15:38. doi: 10.1186/s13148-023-01457-1 (PMC9985232; doi:10.1186/s13148-023-01457-1)
Supplement: Supplementary file 4 — Additional file 4: Table S3. Comparison between our results and other previously reported blood pressure or hypertension-associated differentially methylated genes [file 13148_2023_1457_MOESM4_ESM.docx]

**Additional file 4: Table S3**. Comparison between our results and other previously reported blood pressure or hypertension-associated differentially methylated genes

| **Other studies** | **CpGs in our study** | | | | | |
| --- | --- | --- | --- | --- | --- | --- |
|  | Chromosome | Position (bp) | Coefficient | *p*-value | Ensembl gene ID | HGNC symbol |
| ***SBP*** |  |  |  |  |  |  |
| *Huang Y, Ollikainen M, Muniandy M, Zhang T, van Dongen J, Hao G, van der Most PJ, Pan Y, Pervjakova N, Sun YV, et al. Identification, Heritability, and Relation With Gene Expression of Novel DNA Methylation Loci for Blood Pressure. Hypertension. 2020;76:195-205.* | chr14 | 103,406,521 | -0.052 | 0.011 | ENSG00000198752 | *CDC42BPB* |
|  | chr14 | 103,406,507 | -0.051 | 0.012 | ENSG00000198752 | *CDC42BPB* |
|  | chr14 | 103,400,906 | 0.019 | 0.050 | ENSG00000198752 | *CDC42BPB* |
|  | chr11 | 67,431,665 | -0.009 | 0.022 | ENSG00000132746 | *ALDH3B2* |
|  | chr11 | 67,431,716 | -0.018 | 0.032 | ENSG00000132746 | *ALDH3B2* |
|  | chr11 | 67,431,719 | -0.018 | 0.034 | ENSG00000132746 | *ALDH3B2* |
|  | chr11 | 67,431,473 | -0.006 | 0.042 | ENSG00000132746 | *ALDH3B2* |
|  | chr9 | 124,308,115 | 0.005 | 0.007 | ENSG00000136848 | *DAB2IP* |
|  | chr9 | 124,308,128 | 0.005 | 0.007 | ENSG00000136848 | *DAB2IP* |
|  | chr9 | 124,308,131 | 0.005 | 0.007 | ENSG00000136848 | *DAB2IP* |
|  | chr9 | 124,308,134 | 0.005 | 0.009 | ENSG00000136848 | *DAB2IP* |
|  | chr9 | 124,308,098 | 0.005 | 0.011 | ENSG00000136848 | *DAB2IP* |
|  | chr9 | 124,308,155 | 0.005 | 0.016 | ENSG00000136848 | *DAB2IP* |
|  | chr9 | 124,308,162 | 0.005 | 0.017 | ENSG00000136848 | *DAB2IP* |
|  | chr16 | 87,840,208 | 0.010 | 0.003 | ENSG00000103257 | *SLC7A5* |
|  | chr17 | 40,932,360 | -0.011 | 0.002 | ENSG00000131475 | *VPS25* |
|  | chr17 | 40,932,389 | -0.009 | 0.005 | ENSG00000131475 | *VPS25* |
|  | chr17 | 40,932,338 | -0.011 | 0.008 | ENSG00000131475 | *VPS25* |
|  | chr17 | 40,932,331 | -0.011 | 0.010 | ENSG00000131475 | *VPS25* |
|  | chr17 | 40,932,404 | -0.008 | 0.016 | ENSG00000131475 | *VPS25* |
|  | chr11 | 57,250,123 | 0.010 | 0.020 | ENSG00000149150 | *SLC43A1* |
|  | chr11 | 57,250,101 | 0.009 | 0.028 | ENSG00000149150 | *SLC43A1* |
|  | chr11 | 57,250,160 | 0.010 | 0.029 | ENSG00000149150 | *SLC43A1* |
|  | chr11 | 57,250,175 | 0.010 | 0.037 | ENSG00000149150 | *SLC43A1* |
| *Boström AE, Mwinyi J, Voisin S, Wu W, Schultes B, Zhang K, and Schiöth HB. Longitudinal genome-wide methylation study of Roux-en-Y gastric bypass patients reveals novel CpG sites associated with essential hypertension. BMC Med Genomics. 2016;9:20.* | chr18 | 44,787,548 | 0.008 | 0.001 | ENSG00000215474 | *SKOR2* |
|  | chr18 | 44,787,535 | 0.007 | 0.001 | ENSG00000215474 | *SKOR2* |
|  | chr18 | 44,787,557 | 0.007 | 0.002 | ENSG00000215474 | *SKOR2* |
|  | chr18 | 44,787,560 | 0.007 | 0.002 | ENSG00000215474 | *SKOR2* |
|  | chr18 | 44,787,528 | 0.007 | 0.002 | ENSG00000215474 | *SKOR2* |
|  | chr18 | 44,787,563 | 0.007 | 0.002 | ENSG00000215474 | *SKOR2* |
|  | chr18 | 44,787,525 | 0.007 | 0.002 | ENSG00000215474 | *SKOR2* |
|  | chr18 | 44,787,567 | 0.007 | 0.002 | ENSG00000215474 | *SKOR2* |
|  | chr18 | 44,787,570 | 0.007 | 0.003 | ENSG00000215474 | *SKOR2* |
|  | chr18 | 44,787,574 | 0.007 | 0.004 | ENSG00000215474 | *SKOR2* |
|  | chr18 | 44,787,577 | 0.007 | 0.005 | ENSG00000215474 | *SKOR2* |
|  | chr18 | 44,787,511 | 0.006 | 0.005 | ENSG00000215474 | *SKOR2* |
|  | chr18 | 44,787,586 | 0.007 | 0.009 | ENSG00000215474 | *SKOR2* |
|  | chr18 | 44,787,500 | 0.005 | 0.012 | ENSG00000215474 | *SKOR2* |
|  | chr18 | 44,787,598 | 0.006 | 0.016 | ENSG00000215474 | *SKOR2* |
|  | chr18 | 44,787,603 | 0.006 | 0.021 | ENSG00000215474 | *SKOR2* |
|  | chr18 | 44,787,493 | 0.005 | 0.022 | ENSG00000215474 | *SKOR2* |
|  | chr6 | 16,326,790 | 0.070 | 0.007 | ENSG00000124788 | *ATXN1* |
| *Richard MA, Huan T, Ligthart S, Gondalia R, Jhun MA, Brody JA, Irvin MR, Marioni R, Shen J, Tsai PC, et al. DNA Methylation Analysis Identifies Loci for Blood Pressure Regulation. Am J Hum Genet. 2017;101:888-902.* | chr10 | 81,061,965 | 0.089 | 0.010 | ENSG00000108175 | *ZMIZ1* |
|  | chr11 | 68,550,518 | 0.015 | 0.016 | ENSG00000110090 | *CPT1A* |
|  | chr11 | 68,560,851 | -0.027 | 0.028 | ENSG00000110090 | *CPT1A* |
|  | chr11 | 68,560,835 | -0.027 | 0.031 | ENSG00000110090 | *CPT1A* |
|  | chr11 | 68,550,506 | 0.009 | 0.034 | ENSG00000110090 | *CPT1A* |
| ***DBP*** |  |  |  |  |  |  |
| *Huang Y, Ollikainen M, Muniandy M, Zhang T, van Dongen J, Hao G, van der Most PJ, Pan Y, Pervjakova N, Sun YV, et al. Identification, Heritability, and Relation With Gene Expression of Novel DNA Methylation Loci for Blood Pressure. Hypertension. 2020;76:195-205.* | chr15 | 91,452,485 | 0.088 | 0.028 | ENSG00000196547 | *MAN2A2* |
|  | chr15 | 91,452,525 | 0.094 | 0.030 | ENSG00000196547 | *MAN2A2* |
|  | chr15 | 91,452,498 | 0.087 | 0.031 | ENSG00000196547 | *MAN2A2* |
|  | chr2 | 202,031,003 | 0.054 | 0.036 | ENSG00000003402 | *CFLAR* |
|  | chr2 | 202,031,008 | 0.053 | 0.040 | ENSG00000003402 | *CFLAR* |
|  | chr2 | 202,031,013 | 0.052 | 0.046 | ENSG00000003402 | *CFLAR* |
|  | chr11 | 68,550,518 | 0.015 | 0.016 | ENSG00000110090 | *CPT1A* |
|  | chr11 | 68,560,851 | -0.027 | 0.028 | ENSG00000110090 | *CPT1A* |
|  | chr11 | 68,560,835 | -0.027 | 0.031 | ENSG00000110090 | *CPT1A* |
|  | chr11 | 68,550,506 | 0.009 | 0.034 | ENSG00000110090 | *CPT1A* |
|  | chr9 | 124,308,134 | 0.014 | 0.000 | ENSG00000136848 | *DAB2IP* |
|  | chr9 | 124,308,131 | 0.014 | 0.000 | ENSG00000136848 | *DAB2IP* |
|  | chr9 | 124,308,128 | 0.014 | 0.000 | ENSG00000136848 | *DAB2IP* |
|  | chr9 | 124,308,155 | 0.013 | 0.000 | ENSG00000136848 | *DAB2IP* |
|  | chr9 | 124,308,115 | 0.014 | 0.000 | ENSG00000136848 | *DAB2IP* |
|  | chr9 | 124,308,162 | 0.012 | 0.000 | ENSG00000136848 | *DAB2IP* |
|  | chr9 | 124,308,098 | 0.013 | 0.000 | ENSG00000136848 | *DAB2IP* |
|  | chr9 | 124,308,285 | 0.014 | 0.034 | ENSG00000136848 | *DAB2IP* |
|  | chr16 | 87,840,158 | 0.015 | 0.018 | ENSG00000103257 | *SLC7A5* |
|  | chr16 | 87,840,166 | 0.015 | 0.019 | ENSG00000103257 | *SLC7A5* |
|  | chr16 | 87,840,208 | 0.012 | 0.026 | ENSG00000103257 | *SLC7A5* |
|  | chr1 | 120,192,158 | -0.093 | 0.041 | ENSG00000092621 | *PHGDH* |
| *Boström AE, Mwinyi J, Voisin S, Wu W, Schultes B, Zhang K, and Schiöth HB. Longitudinal genome-wide methylation study of Roux-en-Y gastric bypass patients reveals novel CpG sites associated with essential hypertension. BMC Med Genomics. 2016;9:20.* | chr18 | 44,790,497 | 0.022 | 0.032 | ENSG00000215474 | *SKOR2* |
|  | chr18 | 44,790,503 | 0.022 | 0.032 | ENSG00000215474 | *SKOR2* |
|  | chr18 | 44,790,486 | 0.022 | 0.032 | ENSG00000215474 | *SKOR2* |
|  | chr18 | 44,790,476 | 0.022 | 0.033 | ENSG00000215474 | *SKOR2* |
|  | chr18 | 44,790,465 | 0.021 | 0.037 | ENSG00000215474 | *SKOR2* |
|  | chr18 | 44,790,456 | 0.020 | 0.042 | ENSG00000215474 | *SKOR2* |
| *Richard MA, Huan T, Ligthart S, Gondalia R, Jhun MA, Brody JA, Irvin MR, Marioni R, Shen J, Tsai PC, et al. DNA Methylation Analysis Identifies Loci for Blood Pressure Regulation. Am J Hum Genet. 2017;101:888-902.* | chr10 | 81,061,965 | 0.089 | 0.010 | ENSG00000108175 | *ZMIZ1* |
|  | chr11 | 68,550,518 | 0.015 | 0.016 | ENSG00000110090 | *CPT1A* |
|  | chr11 | 68,560,851 | -0.027 | 0.028 | ENSG00000110090 | *CPT1A* |
|  | chr11 | 68,560,835 | -0.027 | 0.031 | ENSG00000110090 | *CPT1A* |
|  | chr11 | 68,550,506 | 0.009 | 0.034 | ENSG00000110090 | *CPT1A* |
|  | chr1 | 120,192,158 | -0.093 | 0.041 | ENSG00000092621 | *PHGDH* |
