# Supplementary material for: Epigenome-wide association study in Chinese monozygotic twins identifies DNA methylation loci associated with blood pressure
Source: Clin Epigenetics. 2023 Mar 3;15:38. doi: 10.1186/s13148-023-01457-1 (PMC9985232; doi:10.1186/s13148-023-01457-1)
Supplement: Supplementary file 5 — Additional file 5: Table S4. Common ontology enrichments by GREAT tool between systolic blood pressure and diastolic blood pressure [file 13148_2023_1457_MOESM5_ESM.docx]

**Additional file 5: Table S4**. The common ontology enrichments by GREAT tool between systolic blood pressure and diastolic blood pressure

| **Ontology database** | **Term name** |
| --- | --- |
| PANTHER | Cytoskeletal regulation by Rho GTPase |
| PANTHER | Nicotinic acetylcholine receptor signaling pathway |
| PANTHER | Transcription regulation by bZIP transcription factor |
| PANTHER | Metabotropic glutamate receptor group II pathway |
| PANTHER | GABA-B receptor II signaling |
| PANTHER | p53 pathway by glucose deprivation |
| PANTHER | Angiogenesis |
| PANTHER | Inflammation mediated by chemokine and cytokine signaling pathway |
| PANTHER | Endogenous cannabinoid signaling |
| PANTHER | Ornithine degradation |
| PANTHER | Notch signaling pathway |
| PANTHER | Hedgehog signaling pathway |
| PANTHER | Thyrotropin-releasing hormone receptor signaling pathway |
| PANTHER | Nicotine pharmacodynamics pathway |
| PANTHER | Heterotrimeric G-protein signaling pathway-rod outer segment phototransduction |
| PANTHER | Heterotrimeric G-protein signaling pathway-Gq alpha and Go alpha mediated pathway |
| PANTHER | Opioid proopiomelanocortin pathway |
| PANTHER | Gonadotropin-releasing hormone receptor pathway |
| PANTHER | Cortocotropin releasing factor receptor signaling pathway |
| PANTHER | Huntington disease |
| PANTHER | Alzheimer disease-presenilin pathway |
| PANTHER | Insulin/IGF pathway-mitogen activated protein kinase kinase/MAP kinase cascade |
| PANTHER | PI3 kinase pathway |
| PANTHER | General transcription regulation |
| PANTHER | Histamine H1 receptor mediated signaling pathway |
| PANTHER | Oxytocin receptor mediated signaling pathway |
| MSigDB | Transcription Regulation by Methyltransferase of CARM1 |
| MSigDB | Regulation And Function Of ChREBP in Liver |
| MSigDB | Phospholipids as signalling intermediaries |
| MSigDB | fMLP induced chemokine gene expression in HMC-1 cells |
| MSigDB | GATA3 participate in activating the Th2 cytokine genes expression |
| MSigDB | Cytokines and Inflammatory Response |
| MSigDB | Reversal of Insulin Resistance by Leptin |
| MSigDB | Role of MEF2D in T-cell Apoptosis |
| MSigDB | Downregulated of MTA-3 in ER-negative Breast Tumors |
| MSigDB | PKC-catalyzed phosphorylation of inhibitory phosphoprotein of myosin phosphatase |
| MSigDB | Biosynthesis of neurotransmitters |
| MSigDB | NFAT and Hypertrophy of the heart (Transcription in the broken heart) |
| MSigDB | Nuclear Receptors in Lipid Metabolism and Toxicity |
| MSigDB | Deregulation of CDK5 in Alzheimers Disease |
| MSigDB | Thrombin signaling and protease-activated receptors |
| MSigDB | PDGF Signaling Pathway |
| MSigDB | Mechanism of Gene Regulation by Peroxisome Proliferators via PPARa(alpha) |
| MSigDB | Nuclear receptors coordinate the activities of chromatin remodeling complexes and coactivators to facilitate initiation of transcription in carcinoma cells |
| MSigDB | The SARS-coronavirus Life Cycle |
| MSigDB | TACI and BCMA stimulation of B cell immune responses. |
| MSigDB | Control of Gene Expression by Vitamin D Receptor |
| MSigDB | Neuropeptides VIP and PACAP inhibit the apoptosis of activated T cells |
| MSigDB | Adipocytokine signaling pathway |
| MSigDB | Arachidonic acid metabolism |
| MSigDB | Basal cell carcinoma |
| MSigDB | Cell cycle |
| MSigDB | Dorso-ventral axis formation |
| MSigDB | ECM-receptor interaction |
| MSigDB | Focal adhesion |
| MSigDB | Glioma |
| MSigDB | Glutathione metabolism |
| MSigDB | Glycosylphosphatidylinositol(GPI)-anchor biosynthesis |
| MSigDB | Hedgehog signaling pathway |
| MSigDB | Huntington's disease |
| MSigDB | Inositol phosphate metabolism |
| MSigDB | Insulin signaling pathway |
| MSigDB | Lysosome |
| MSigDB | MAPK signaling pathway |
| MSigDB | Maturity onset diabetes of the young |
| MSigDB | Notch signaling pathway |
| MSigDB | Pathogenic Escherichia coli infection |
| MSigDB | Proximal tubule bicarbonate reclamation |
| MSigDB | Regulation of actin cytoskeleton |
| MSigDB | T cell receptor signaling pathway |
| MSigDB | Taste transduction |
| MSigDB | Tight junction |
| MSigDB | Type II diabetes mellitus |
| MSigDB | VEGF signaling pathway |
| MSigDB | Vibrio cholerae infection |
| MSigDB | Wnt signaling pathway |
| MSigDB | ALK2 signaling events |
| MSigDB | amb2 Integrin signaling |
| MSigDB | ATM pathway |
| MSigDB | Ceramide signaling pathway |
| MSigDB | C-MYB transcription factor network |
| MSigDB | Validated transcriptional targets of deltaNp63 isoforms |
| MSigDB | E-cadherin signaling in the nascent adherens junction |
| MSigDB | Stabilization and expansion of the E-cadherin adherens junction |
| MSigDB | Signaling events mediated by HDAC Class I |
| MSigDB | Notch-mediated HES/HEY network |
| MSigDB | FOXA2 and FOXA3 transcription factor networks |
| MSigDB | IL8- and CXCR2-mediated signaling events |
| MSigDB | Alpha4 beta1 integrin signaling events |
| MSigDB | LKB1 signaling events |
| MSigDB | mTOR signaling pathway |
| MSigDB | p53 pathway |
| MSigDB | Class I PI3K signaling events |
| MSigDB | Presenilin action in Notch and Wnt signaling |
| MSigDB | RAC1 signaling pathway |
| MSigDB | Glucocorticoid receptor regulatory network |
| MSigDB | RhoA signaling pathway |
| MSigDB | Regulation of RhoA activity |
| MSigDB | Visual signal transduction: Rods |
| MSigDB | RXR and RAR heterodimerization with other nuclear receptor |
| MSigDB | Regulation of nuclear SMAD2/3 signaling |
| MSigDB | Syndecan-1-mediated signaling events |
| MSigDB | JNK signaling in the CD4+ TCR pathway |
| MSigDB | Neurotrophic factor-mediated Trk receptor signaling |
| MSigDB | Thromboxane A2 receptor signaling |
| MSigDB | Urokinase-type plasminogen activator (uPA) and uPAR-mediated signaling |
| MSigDB | Abortive elongation of HIV-1 transcript in the absence of Tat |
| MSigDB | Acetylcholine Binding And Downstream Events |
| MSigDB | Activation of Chaperone Genes by XBP1(S) |
| MSigDB | Acyl chain remodelling of PC |
| MSigDB | Acyl chain remodelling of PG |
| MSigDB | ADP signalling through P2Y purinoceptor 1 |
| MSigDB | ADP signalling through P2Y purinoceptor 12 |
| MSigDB | AKT phosphorylates targets in the cytosol |
| MSigDB | Aquaporin-mediated transport |
| MSigDB | Base Excision Repair |
| MSigDB | Base-free sugar-phosphate removal via the single-nucleotide replacement pathway |
| MSigDB | Basigin interactions |
| MSigDB | Biosynthesis of the N-glycan precursor (dolichol lipid-linked oligosaccharide, LLO) and transfer to a nascent protein |
| MSigDB | Caspase-mediated cleavage of cytoskeletal proteins |
| MSigDB | Cell Cycle, Mitotic |
| MSigDB | Cell death signalling via NRAGE, NRIF and NADE |
| MSigDB | Chylomicron-mediated lipid transport |
| MSigDB | Class B/2 (Secretin family receptors) |
| MSigDB | Costimulation by the CD28 family |
| MSigDB | CTLA4 inhibitory signaling |
| MSigDB | Beta-catenin phosphorylation cascade |
| MSigDB | Destabilization of mRNA by KSRP |
| MSigDB | Downregulation of SMAD2/3:SMAD4 transcriptional activity |
| MSigDB | Downregulation of TGF-beta receptor signaling |
| MSigDB | Downstream signal transduction |
| MSigDB | Elongation arrest and recovery |
| MSigDB | Endosomal Sorting Complex Required For Transport (ESCRT) |
| MSigDB | Facilitative Na+-independent glucose transporters |
| MSigDB | Factors involved in megakaryocyte development and platelet production |
| MSigDB | Formation of RNA Pol II elongation complex |
| MSigDB | Formation of the HIV-1 Early Elongation Complex |
| MSigDB | Formation of tubulin folding intermediates by CCT/TriC |
| MSigDB | G alpha (12/13) signalling events |
| MSigDB | G beta:gamma signalling through PI3Kgamma |
| MSigDB | G beta:gamma signalling through PLC beta |
| MSigDB | G-protein activation |
| MSigDB | G-protein beta:gamma signalling |
| MSigDB | Generation of second messenger molecules |
| MSigDB | Generic Transcription Pathway |
| MSigDB | Glucagon signaling in metabolic regulation |
| MSigDB | Glucagon-type ligand receptors |
| MSigDB | Glycerophospholipid biosynthesis |
| MSigDB | HDL-mediated lipid transport |
| MSigDB | Hemostasis |
| MSigDB | Highly calcium permeable postsynaptic nicotinic acetylcholine receptors |
| MSigDB | Inhibition of Insulin Secretion by Adrenaline/Noradrenaline |
| MSigDB | Integration of energy metabolism |
| MSigDB | Interaction between L1 and Ankyrins |
| MSigDB | Lipid digestion, mobilization, and transport |
| MSigDB | Lipoprotein metabolism |
| MSigDB | Loss of Nlp from mitotic centrosomes |
| MSigDB | Metabolism of proteins |
| MSigDB | Metabolism of vitamins and cofactors |
| MSigDB | Mitotic G2-G2/M phases |
| MSigDB | Mitotic Prometaphase |
| MSigDB | mRNA Processing |
| MSigDB | mRNA Splicing |
| MSigDB | mRNA Splicing - Minor Pathway |
| MSigDB | NCAM signaling for neurite out-growth |
| MSigDB | NCAM1 interactions |
| MSigDB | NF-kB is activated and signals survival |
| MSigDB | NRAGE signals death through JNK |
| MSigDB | p75 NTR receptor-mediated signalling |
| MSigDB | PD-1 signaling |
| MSigDB | Platelet activation, signaling and aggregation |
| MSigDB | Platelet sensitization by LDL |
| MSigDB | Post-chaperonin tubulin folding pathway |
| MSigDB | Prefoldin mediated transfer of substrate to CCT/TriC |
| MSigDB | Presynaptic nicotinic acetylcholine receptors |
| MSigDB | Processing of Capped Intron-Containing Pre-mRNA |
| MSigDB | Prostacyclin signalling through prostacyclin receptor |
| MSigDB | Protein folding |
| MSigDB | Recruitment of mitotic centrosome proteins and complexes |
| MSigDB | Recruitment of NuMA to mitotic centrosomes |
| MSigDB | Regulation of beta-cell development |
| MSigDB | Regulation of gene expression in beta cells |
| MSigDB | Regulation of Insulin-like Growth Factor (IGF) Activity by Insulin-like Growth Factor Binding Proteins (IGFBPs) |
| MSigDB | Regulation of Insulin Secretion |
| MSigDB | Regulation of Insulin Secretion by Glucagon-like Peptide-1 |
| MSigDB | Regulation of Water Balance by Renal Aquaporins |
| MSigDB | Resolution of AP sites via the multiple-nucleotide patch replacement pathway |
| MSigDB | Resolution of AP sites via the single-nucleotide replacement pathway |
| MSigDB | Response to elevated platelet cytosolic Ca2+ |
| MSigDB | RNA Polymerase II Pre-transcription Events |
| MSigDB | RORA Activates Circadian Expression |
| MSigDB | Signal amplification |
| MSigDB | Signaling by FGFR3 mutants |
| MSigDB | Signaling by PDGF |
| MSigDB | Signaling by TGF-beta Receptor Complex |
| MSigDB | Synthesis of substrates in N-glycan biosythesis |
| MSigDB | Synthesis, Secretion, and Inactivation of Glucagon-like Peptide-1 (GLP-1) |
| MSigDB | Termination of O-glycan biosynthesis |
| MSigDB | TGF-beta receptor signaling activates SMADs |
| MSigDB | Thrombin signalling through proteinase activated receptors (PARs) |
| MSigDB | Thromboxane signalling through TP receptor |
| MSigDB | Transcriptional activity of SMAD2/SMAD3:SMAD4 heterotrimer |
| MSigDB | Transcriptional Regulation of White Adipocyte Differentiation |
| MSigDB | Transport of vitamins, nucleosides, and related molecules |
| MSigDB | Unwinding of DNA |
| MSigDB | Voltage gated Potassium channels |
| MSigDB | Xenobiotics |
| MSigDB | YAP1- and WWTR1 (TAZ)-stimulated gene expression |
| MSigDB | Regulation of the actin cytoskeleton |

**Note**: DBP, diastolic blood pressure; SBP, systolic blood pressure
