# Supplementary material for: Epigenome-wide association study in Chinese monozygotic twins identifies DNA methylation loci associated with blood pressure
Source: Clin Epigenetics. 2023 Mar 3;15:38. doi: 10.1186/s13148-023-01457-1 (PMC9985232; doi:10.1186/s13148-023-01457-1)
Supplement: Supplementary file 6 — Additional file 6: Table S5. The results of validation analysis for the CpGs mapped to COL5A1 on systolic blood pressure [file 13148_2023_1457_MOESM6_ESM.docx]

**Additional file 6: Table S5**. The results of validation analysis for the CpGs mapped to *COL5A1* on systolic blood pressure

| **CpG No.** | **Chromosome** | **Position (bp)** | **Discovery** | |  | **Validation** | | | | |
| --- | --- | --- | --- | --- | --- | --- | --- | --- | --- | --- |
|  |  |  | Coefficient | *P*-value |  | *p*-value of comparison between groups |  | Coefficient | *P*-value | OR (95% CI) |
| 1 | Chr9 | 137674004 | -0.010 | 1.574E-02 |  | 0.355 |  | -0.150 | 0.292 | 0.861 (0.648-1.137) |
| 2 | Chr9 | 137673999 | -0.010 | 2.040E-02 |  | 0.388 |  | -0.139 | 0.331 | 0.870 (0.654-1.152) |
| 3 | Chr9 | 137673952 | -0.007 | 1.275E-02 |  | 0.727 |  | -0.104 | 0.608 | 0.901 (0.603-1.343) |
| 4 | Chr9 | 137673928 | -0.008 | 7.284E-04 |  | 0.052 |  | -0.269 | 0.150 | 0.764 (0.526-1.099) |
| 5^*^ | Chr9 | 137673907 | -0.009 | 3.066E-05 |  | 0.044 |  | -0.439 | 0.048 | 0.644 (0.412-0.989) |
| 6 | Chr9 | 137673895 | -0.009 | 2.971E-05 |  | 0.014 |  | -0.012 | 0.893 | 0.988 (0.823-1.182) |
| 7 | Chr9 | 137673888 | -0.009 | 4.699E-05 |  | 0.281 |  | -0.130 | 0.121 | 0.878 (0.743-1.035) |
| 8 | Chr9 | 137673850 | -0.010 | 4.731E-04 |  | 0.132 |  | -0.224 | 0.189 | 0.800 (0.568-1.112) |

Note: * The CpG significantly associated with hypertension
