# Supplementary material for: Epigenome-wide association study in Chinese monozygotic twins identifies DNA methylation loci associated with blood pressure
Source: Clin Epigenetics. 2023 Mar 3;15:38. doi: 10.1186/s13148-023-01457-1 (PMC9985232; doi:10.1186/s13148-023-01457-1)
Supplement: Supplementary file 7 — Additional file 7: Table S6. The results of validation analysis for the CpGs mapped to WNT3A on diastolic blood pressure [file 13148_2023_1457_MOESM7_ESM.docx]

**Additional file 7: Table S6**. The results of validation analysis for the CpGs mapped to *WNT3A* on diastolic blood pressure

| **CpG No.** | **Chromosome** | **Position (bp)** | **Discovery** | |  | **Validation** | | | | |
| --- | --- | --- | --- | --- | --- | --- | --- | --- | --- | --- |
|  |  |  | Coefficient | *P*-value |  | *p*-value of comparison between groups ^#^ |  | Coefficient | *P*-value | OR (95% CI) |
| 1^*^ | chr1 | 228195277 | 0.028 | 5.764E-08 |  | - |  | - | - | - |
| 2^#^ | chr1 | 228195289 | 0.029 | 1.291E-07 |  | 0.005 |  | 0.484 | 0.003 | 1.622 (1.188-2.254) |
| 3^#^ | chr1 | 228195292 | 0.029 | 1.633E-07 |  | 0.005 |  | 0.485 | 0.003 | 1.625 (1.193-2.251) |
| 4^*^ | chr1 | 228195260 | 0.029 | 2.857E-07 |  | - |  | - | - | - |
| 5^#^ | chr1 | 228195243 | 0.029 | 1.988E-05 |  | 0.007 |  | 0.156 | 0.022 | 1.169 (1.024-1.340) |

**Note**: ^*^ The CpGs were not detected in the validation experiment.

^#^ The CpG significantly associated with hypertension
