# Supplementary figures and images for: Epigenome-wide association study in Chinese monozygotic twins identifies DNA methylation loci associated with blood pressure
Source: Clin Epigenetics. 2023 Mar 3;15:38. doi: 10.1186/s13148-023-01457-1 (PMC9985232; doi:10.1186/s13148-023-01457-1)

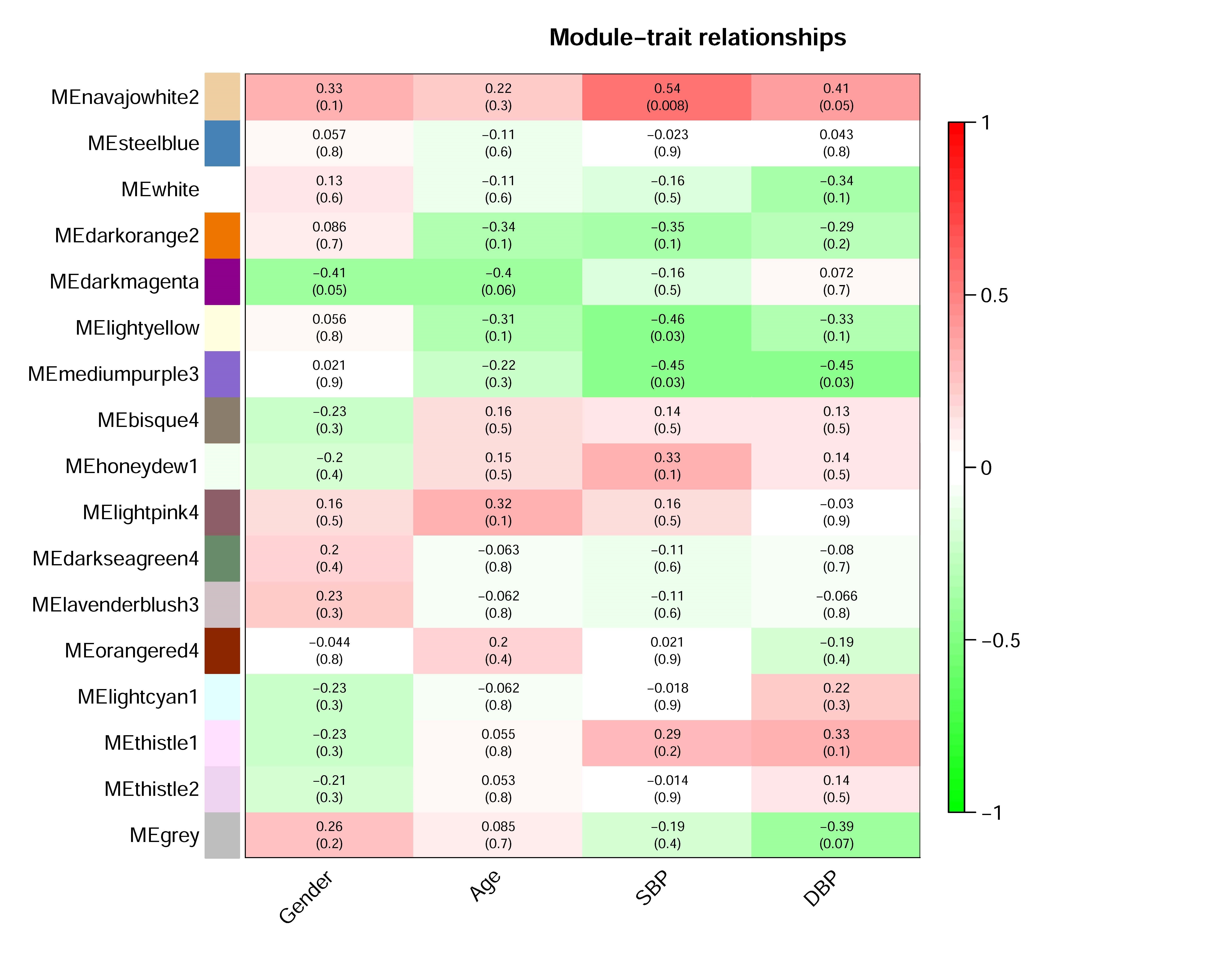

Supplement: Supplementary file 8 — Additional file 8: Fig. S2. Relationships of consensus module eigengenes and external trait of blood pressure. Numbers in the table report the correlations with the p-values printed in parentheses. The table is color coded by correlation according to the color legend. [file 13148_2023_1457_MOESM8_ESM.jpg]
