# Supplementary material for: Epigenome-wide association study in Chinese monozygotic twins identifies DNA methylation loci associated with blood pressure
Source: Clin Epigenetics. 2023 Mar 3;15:38. doi: 10.1186/s13148-023-01457-1 (PMC9985232; doi:10.1186/s13148-023-01457-1)
Supplement: Supplementary file 9 — Additional file 9: Table S7. Common enrichment terms for blood pressure between methylation analysis and weighted gene co-expression network analysis [file 13148_2023_1457_MOESM9_ESM.docx]

**Additional file 9: Table S7**. Common enrichment terms for blood pressure between methylation analysis and weighted gene co-expression network analysis

| **Trait** | **Module in WGCNA** | **Ontology** | **ID** | **Desciption** |
| --- | --- | --- | --- | --- |
| ***SBP*** |  |  |  |  |
|  | mediumpurple3 | GO Molecular Function | GO:0019899 | enzyme binding |
|  |  | GO Molecular Function | GO:0004252 | serine-type endopeptidase activity |
|  |  | GO Molecular Function | GO:0003899 | DNA-directed RNA polymerase activity |
|  |  | GO Molecular Function | GO:0030506 | ankyrin binding |
|  |  | GO Molecular Function | GO:0005245 | voltage-gated calcium channel activity |
|  |  | GO Molecular Function | GO:0008137 | NADH dehydrogenase (ubiquinone) activity |
|  |  | GO Molecular Function | GO:0004844 | uracil DNA N-glycosylase activity |
|  |  | GO Molecular Function | GO:0005088 | Ras guanyl-nucleotide exchange factor activity |
|  |  | GO Molecular Function | GO:0019104 | DNA N-glycosylase activity |
|  |  | GO Molecular Function | GO:0005515 | protein binding |
|  |  | GO Molecular Function | GO:0003735 | structural constituent of ribosome |
|  |  | GO Molecular Function | GO:0051087 | chaperone binding |
|  |  | GO Molecular Function | GO:0017070 | U6 snRNA binding |
|  |  | GO Biological Process | GO:0007274 | neuromuscular synaptic transmission |
|  |  | MSigDB Pathway-KEGG | - | Base excision repair |
|  |  | MSigDB Pathway-KEGG | - | Huntington's disease |
|  |  | MSigDB Pathway-KEGG | - | PPAR signaling pathway |
| ***DBP*** |  |  |  |  |
|  | mediumpurple3 | GO Molecular Function | GO:0030506 | ankyrin binding |
|  |  | GO Molecular Function | GO:0003735 | structural constituent of ribosome |
|  |  | GO Molecular Function | GO:0004252 | serine-type endopeptidase activity |
|  |  | GO Molecular Function | GO:0005245 | voltage-gated calcium channel activity |
|  |  | GO Molecular Function | GO:0034617 | tetrahydrobiopterin binding |
|  |  | GO Molecular Function | GO:0015464 | acetylcholine receptor activity |
|  |  | GO Molecular Function | GO:0030898 | actin-dependent ATPase activity |
|  |  | GO Molecular Function | GO:0019104 | DNA N-glycosylase activity |
|  |  | GO Molecular Function | GO:0004844 | uracil DNA N-glycosylase activity |
|  |  | GO Molecular Function | GO:0005515 | protein binding |
|  |  | GO Molecular Function | GO:0015631 | tubulin binding |
|  |  | GO Molecular Function | GO:0005088 | Ras guanyl-nucleotide exchange factor activity |
|  |  | GO Molecular Function | GO:0017070 | U6 snRNA binding |
|  |  | GO Molecular Function | GO:0019843 | rRNA binding |
|  |  | GO Biological Process | GO:0036297 | interstrand cross-link repair |
|  |  | GO Biological Process | GO:0070126 | mitochondrial translational termination |
|  |  | GO Biological Process | GO:0045879 | negative regulation of smoothened signaling pathway |
|  |  | MSigDB Pathway-KEGG | - | Oxidative phosphorylation |
|  |  | MSigDB Pathway-KEGG | - | Huntington's disease |
